# Espy’s Lectures on Meteorology

**Published:** 1840

**Authors:** 


### PROFESSOR ESPY'S LECTURES ON METEOROLOGY.

The West has lately been visited by this distinguished Meteorologist, who lectured in New Orleans, St. Louis, Louisville, Cincinnati and Pittsburg. Mr. Espy's main object is, by the aid of diagrams and experiments, to explain to the people, or at least the intelligent and inquiring portions of them, his philosophy of rain and storms; that they may be incited to make such observations, as will demonstrate the truth of his theory. We had not the advantage of hearing more than two of his prelections. The *first*, comprising his data, was uncommonly interesting to the whole audience; the *second* seemed to us rather recondite for the popular mind, and had, moreover, a minuteness of detail, for which a mixed audience is not in general prepared. At some future time, we hope, to be able to present a full view of Mr. Espy's system of meteorology, and shall, therefore, at present limit ourselves to the annunciation of his great principle. It is, that caloric, acting in and upon the atmosphere, is the efficient cause of dew, frost, fog, haze, clouds, rain, hail, snow, winds and tornadoes. We may cite one example: When the air over any particular place becomes heated, ascends, and, under diminished pressure expands, this expansion by cooling it condenses its moisture and forms clouds, which fall in rain—the winds meantime blowing towards the spot from all directions. Such being the origin of rain, it is certainly, *in theory*, possible to produce it by artificial heat. It was the annunciation of this *possibility*, as a *practicability*, that startled so many minds, and for some time brought the ingenious author into philosophic discredit, with those who did not understand the principles from which his conclusion was deduced. Wherever Mr. Espy has lectured he has attracted much attention to this beautiful department of natural science, and made many converts. We understand that it is his design to attend the meetings of the British Association at Glasgow the ensuing autumn. D.

